# Green Transforming Metallurgical Residue into Alkali-Activated Silicomanganese Slag-Based Cementitious Material as Photocatalyst

**DOI:** 10.3390/ma11091773

**Published:** 2018-09-19

**Authors:** Yao Jun Zhang, Pan Yang He, Hao Chen, Li Cai Liu

**Affiliations:** 1 College of Materials and Mineral Resources, Xi’an University of Architecture and Technology, Xi’an 710055, China; hepanyang@live.xauat.edu.cn (P.Y.H.); chenhao@live.xauat.edu.cn (H.C.); liulicai@163.com (L.C.L.); 2 State Key Laboratory of Green Building in Western China, Xi’an University of Architecture and Technology, Xi’an 710055, China

**Keywords:** silicomanganese slag, alkali-activated cementitious material, electrical conductivity

## Abstract

Silicomanganese slag is a solid waste in metallurgical industry and can be transformed into an alkali-activated silicomanganese slag-based cementitious-material (ASSC) for the first time. The ASSC shows quite low electro-conductivity and can be raised dramatically by incorporated carbon black (CB) in the matrix of ASSC to create an electro-conductive alkali-activated silicomanganese slag-based cementitious-composite (EASSC), served as a low cost and environmentally-friendly photocatalyst for the removal of dye pollutant in the paper. The interrelationships of mechanical, optical, electroconductive, microstructural, and photocatalytic properties are evaluated. The network of CB plays a critical role in the electron transfers. The electrical conductivity of EASSC doped 4.5% CB drastically increases by 594.2 times compared to that of ASSC. The FESEM, XRD, and XPS results indicated that the EASSC with mean grain size about 50 nm is composed of amorphous calcium silicate hydrate (CSH), alabandite (α-MnS) and CB. The UV–vis DRS and PL exhibit that the absorption edges of electro-conductive alkali-activated silicomanganese slag-based cementitious-composite EASSC samples are gradually blue-shifted and the photoluminescence intensities progressively decrease with increasing CB content. The activities of photocatalytic degradation of basic violet 5BN dye are positive correlated to the electro-conductivities. The separation efficiency of photo-generated electron-hole pairs is enhanced due to the electron transfers from α-MnS to the network of CB. The photocatalytic degradation of dye pollutant belongs to the second order kinetics via a reaction mechanism of superoxide radical (•O_2_^−^) intermediate.

## 1. Introduction

Much more attention has been focused on utilization of industrial by-products and wastes recycling due to the growing scarce resources and eco-environmental concerns. Silicomanganese slag (SS) is a by-product with higher manganese content generated by the smelting manganese ferroalloy plant for making manganese-steel pig-iron. In the recent three years from 2014 to 2016, the output of silicon-manganese alloy is about 35.7 million tons in China [1]. According to the statistics from the enterprises, about 1.2–1.3 tons silicon manganese slag will be discharged to produce per ton silicon-manganese alloy. A total of about 46.41 million tons of SS is emitted, but very few have been recycled owing to its low activity as a cementitious material [2]. With the rapid development of ferroalloy smelting industry in China, the emission of SS is gradually increased year by year and puts the pressures on the sustainable development of ecological environment and economy. Therefore, the low cost and eco-friendly utilization of SS as a resource has become a big society issue and attracted much attention. In absence of pozzolanic activity, SS was often used as an admixture at the mass ratio of 5–15% in Portland cements [3], road base material such as rigid and flexible pavements, rail track, embankment, and backfill material [4]. In order to improve the reactivity, Kumar et al. reported that the SS was activated by mechanical methods of eccentric vibratory and attrition mill, and the activated SS was used to develop an alkali-activated cementitious material [5]. Up to now, limited information was available on alkali-activated silicomanganese slag-based cementitious material utilization in high value addition.

Carbon black has been widely used in industries due to its high specific area, high mechanical strength, and excellent thermal and electrical conductivity. Carbon black consists of primary particles with average particle sizes from a few nanometers to about 100 nm depending on the preparation techniques. The structure inside primary particles mainly contains polycrystalline and amorphous phases, such as onion with ordered graphitic layer structures, plane graphitic structures with randomly oriented basic structural units, and amorphous phase containing single graphene layers with a straight or bent structure randomly oriented [6]. The structures of the carbon black involving small clusters, nanoparticles, and macroscopic aggregates have a strong influence on their optical properties, apart from the shape and agglomeration state of the primary particles [6,7]. The shape and position of the UV feature in the optical extinction at about 217.5 nm depends on the internal structure of the particles [8,9]. The UV π-π * absorption feature of the carbon blacks varies from 196 nm to 265 nm according to the state of bending of the graphene layers in the subunits of particles and the dimensions of the plane graphitic microcrystallites [10]. The carbon black particle shows a higher conductivity, and E_g_ values in the order of 0.8–1.5 eV rely on the particle sizes [7]. If the electro-conductivity of carbon black is coupled with the alkali-activated solid waste-based colloidal material, it will create a new electro-conductively cementitious material. 

In the present study, an alkali-activated silicomanganese slag-based cementitious-material (ASSC) and an electro-conductive alkali-activated silicomanganese slag-based cementitious-composite (EASSC) was prepared by incorporating carbon black in the matrix of ASSC for the first time. The purposes attempt to (1) develop a novel electro-conductive alkali-activated silicomanganese slag-based cementitious material, (2) realize low cost, environmentally friendly and recycle utilization of silicomanganese slag on a large scale, and (3) establish a new relationship between electro-conductivity and removal of dye pollutant. It could bring about technical, economical, and mainly environmental benefits through high-value resource utilization of silicomanganese slag. The main idea wants to sufficiently utilize electro-conductive network of carbon black for transmission photo-generated electron of α-MnS semiconductor derived from silicomanganese slag so as to improve the separation efficiency of photo-generated electron-hole pairs and to enhance the degradation rate of dye pollutant.

## 2. Materials and Methods

### 2.1. Materials

Silicomanganese slag was obtained from Mianxian Steel Company, Hanzhong, China, and was ground to a Blaine specific surface area of 532 m^2^·kg^−1^ and a density of 2.78 g·cm^−3^. The chemical composition was shown in Table 1. A chemical activator, NaOH (A.P.), was purchased from Sinopharm Chemical Reagent Co., Ltd., Xi’an, China, and the electroconductive carbon black was obtained from Tianjin Baochi Chemical Co., Ltd., Tianjin, China.

### 2.2. Preparation of EASSC

EASSC sample was prepared by a blend of silicomanganese slag (SS), activator (NaOH), carbon black (CB) and water at a mass ratio of 1:0.04:0.015:0.35. Put SS and CB in a slurry mixer and then add an aqueous solution of NaOH activator with stirring for 2 min. The paste was put into 31.5 × 31.5 × 50 mm^3^ triplicate stainless steel mold by vibro-casting, and then four electrodes were inserted. The paste was sealed in a polyethylene film bag to cure at 80 °C for 6 h, and then kept at curing room for 18 h. After demolded, the paste sample was cured at curing room with a temperature of 20 °C and 95% relative humidity for different days. The sample incorporated 1.5 wt % carbon black was assigned as 1.5EASSC (Electroconductively Alkali-activated Silicomanganese Slag-based cementitious-composite by doped 1.5 wt % carbon black). According to similar experimental procedures, the samples doped 3.5 wt % and 4.5 wt % carbon black were prepared and designated as 3.5EASSC and 4.5EASSC, respectively. The electrical conductivity of samples was detected by four-electrode method. The EASSC sample was crushed and screened to obtain the photocatalyst with particle size distribution in the range of 0.16–0.315 mm.

### 2.3. Characterization of EASSC

Elemental analysis was carried out on an X-ray fluorescence (XRF) analyzer (S4 Pioneer, Bruker, Karlsruhe, Germany). Compressive strength was measured on a YAW-300 automatic pressure testing machine (YAW-300, Shaoxing, China) at a loading speed of 2.4 kN·s^−1^. The morphology observation was performed on a field emission scanning electron microscopy (FESEM) (S-4800, Hitachi, Tokyo, Japan). X-ray diffraction patterns were measured on an X-ray diffractometer (D/MAX-2400, Rigaku, Tokyo, Japan) equipped with a rotation anode using a CuKα radiation with 0.02° of 2θ step intervals and working electric current of 40 mA and voltage of 40 kV. UV-vis diffuse reflectance spectra (UV-vis DRS) were recorded on a UV-vis-NIR spectrophotometer (Cary 5000, Agilent, Santa Clara, CA, USA) equipped with an integrating sphere using BaSO_4_ as a reference. Surface composition and chemical states were taken on an X-ray photoelectron spectroscopy (XPS) (AXIS SUPRA, Shimadzu, Tokyo, Japan) using AlKα (1486.6 eV) as an excitation source.

### 2.4. Evaluation of Photocatalytic Activity

The photocatalytic activities of samples were evaluated by degradation the basic violet 5BN dye, in which the initial absorbance A_0_ was measured at the maximum absorption wavelength of 580 nm. 0.8 g of photocatalyst sample was put into the beaker that contains 100 mL aqueous solution of 4 mg·L^−1^ basic violet 5BN dye. After the adsorption-desorption equilibrium of dye molecules on solid sample achieved in the dark room, the solution suspending solid particles was irradiated by a UV-lamp (365 nm, 18 W) for different times under magnetic stirring at room temperature and the absorbance A_t_ was detected. The photocatalytic degradation efficiency (PDE) of dye was calculated using Equation (1).

PDE = [(C_0_ − C_t_)/C_0_] × 100% = [(A_0_ − A_t_)/A_0_] × 100% (1)

## 3. Results and Discussions

### 3.1. Interrelationship of Electrical Conductivity, Mechanical Strength and Microstructure

Table 1 lists the oxide composition of SS and 4.5EASSC samples. The electrical conductivity at different cuing ages and the compressive strength at curing age of 28 days for all of samples are summarized in Table 2.

It can be found from Table 2 that the electrical conductivity for each sample decreases slowly at curing ages of 3 days, 7 days, and 14 days, and the value approximately tends to be stable at curing age of 28 days, ascribing to that the ionic electrical conductivity gradually becomes stable when free water transforms into combined water during the hydration process of silicomanganese slag. The electrical conductivity is about 0.0005 (S·m^−1^) for the ASSC sample. When 1.5 wt % carbon black is incorporated into the ASSC, the stable electrical conductivity is about 0.0006 (S·m^−1^) for 1.5EASSC sample, 0.1155 (S·m^−1^) for 3.5EASSC sample and 0.2976 (S·m^−1^) for 4.5EASSC sample. The electrical conductivity increases by 0.20, 230, and 594.2 times compared to that of the ASSC sample at curing age of 28 days, respectively. The electrical conductivity of EASSC sample drastically increases with increasing of carbon black content, indicating that the carbon black particles overlap each other to form a cross-linking electro-conductive network, while carbon black conducts electric current in the plane of each covalently bonded sheet due to the delocalization of one outer electron in each atom to form π-cloud [11]. From Table 2, it can be observed that even though there is a progressive decrease for samples in compressive strengths from 45.6 MPa to 13.0 MPa, it meets the requirement of self-supporting strength of solid catalyst.

**Figure 1:**
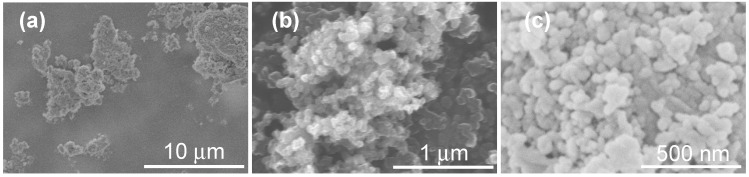
FESEM photographs of (**a**) silicomanganese slag (SS); (**b**) carbon black (CB); (**c**) 4.5EASSC.

FESEM photographs of silicomanganese slag (SS), carbon black (CB), and 4.5EASSC samples are depicted in Figure 1. The irregular particles of SS sample vary in the size from about 1 μm to 10 μm. The spherical granules of carbon black in mean size of about 60 nm appear to be greatly aggregated. The morphology of 4.5EASSC sample consists of homogeneous particles in the size of about 50 nm and the microstructure is quite dense because the carbon black fills in the interstitial sites to decrease the porosity of ASSC. Generally, densification is one of the most significant methods to improve electrical conductivity of materials. Wagner et al. put forward a linear relationship to describe the porosity and electrical conductivity for polycrystalline graphite [12]. Some researchers developed models to formulate the relationship between porosity and electronic properties [13].

**Figure 2:**
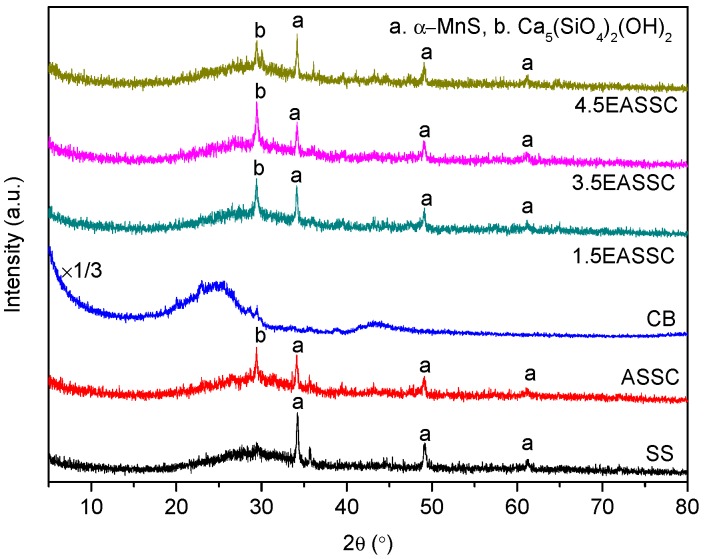
XRD patterns of samples.

Figure 2 displays the XRD patterns of samples. The SS pattern appears a diffused wide band which extends from 20° to 38° and accompanies the hump at approximately about 30° of 2θ, in which the diffraction pattern has the characteristics of the short-range order of the C–S–A–M (CaO–SiO_2_–Al_2_O_3_–MgO) glass structure [14]. The peaks placed at 34.19°, 49.14°, and 61.22° of 2θ corresponding to the crystal faces of (200), (220), and (222) are identified as alabandite (α-MnS, JCPDS 72-1534). There are two peaks at 24.5° and 43.6° in the CB pattern belonging to the feature of graphitic microcrystalline structure [15,16]. When SS, CB and aqueous solution of NaOH are mixed under curing temperature of 80 °C for 6 h and then at curing room for 28 days, the amorphous SiO_2_, CaO and H_2_O react with NaOH to generate calcium silicate hydroxide (CSH) (Ca_5_(SiO_4_)_2_(OH)_2_, JCPDS No. 84-0148) with the diffraction peak at 29.39° of 2θ in the patterns of 1.5EASSC, 3.5EASSC, and 4.5EASSC samples, while the spherical granules of carbon black are evenly dispersed in the phase of C–S–H and concatenate each other into electro-conductive network to exhibit excellent electrical conductivity, as described in Table 2.

### 3.2. Spectral Features of EASSC

**Figure 3:**
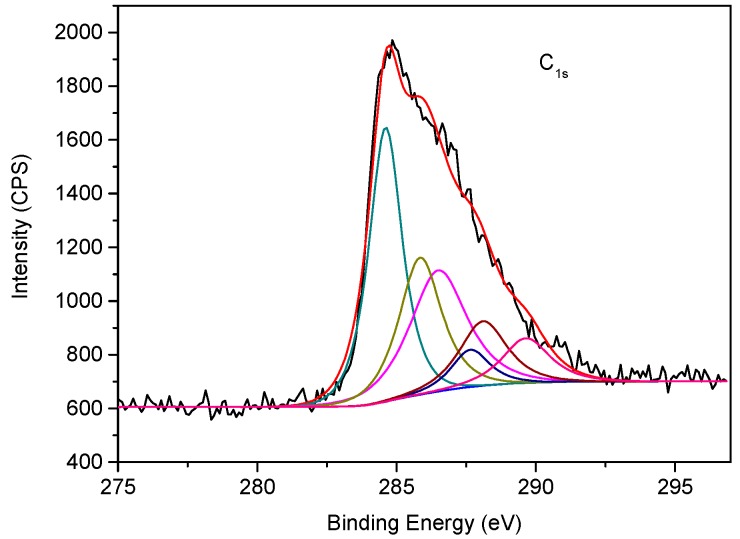
High-resolution C1s XPS spectra of 4.5EASSC sample.

Figure 3 shows the high resolution C1s XPS spectrum of 4.5EASSC sample. The electron binding energy of C1s in carbon black from 280 eV to 295 eV, which is composed of six kinds of chemical oxygen-containing functional groups, corresponding to C–C at 284.6 eV, C–OH at 286.1 eV, C–O at 286.5 eV, C=O at 287.6 eV, O–C=O at 288.1 eV, and COOH at 289.6 eV [17]. The formations of different carbon-oxygen bonds probably results from two main reasons, one is the carbon black is exposed to air to form some carbon-oxygen groups, the other is the granular surface of carbon black is corroded by alkaline solution of activator and then is oxidized by air during the long curing time of 28 days.

**Figure 4:**
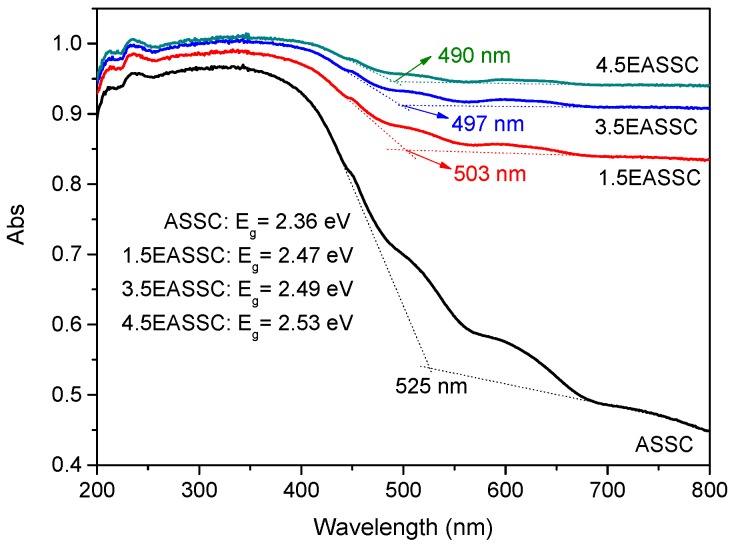
UV–vis diffuse reflectance spectra of specimens.

Figure 4 shows the UV–vis diffuse reflectance spectra of samples. The ASSC sample exhibits a lowest intensity of spectrum with an absorption edge of 525 nm. The samples doped with carbon black possess higher intensities corresponding to the absorption edges of 503 nm for 1.5EASSC, 497 nm for 3.5EASSC, and 490 nm for 4.5EASSC, respectively. Meanwhile, the absorption edges gradually blue shift with increasing carbon black content due to literally darkening of samples. The shortest absorption edge of 490 nm for 4.5EASSC is still in visible region.

**Figure 5:**
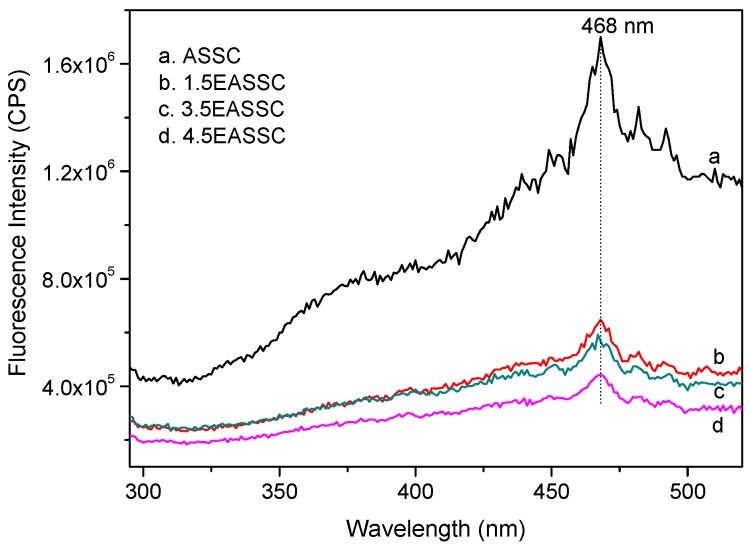
Photoluminescence spectra of samples (excitation wavelength of 280 nm).

Figure 5 depicts the photoluminescence spectra of samples. When the samples are excited by 280 nm wavelength from an emitter of xenon lamp, all samples appear at the biggest photo-fluorescence peak at 468 nm. The ASSC sample displays the strongest photoluminescence curve, demonstrating that the photo-generated electron and hole are easy recombination. The photoluminescence intensities for the samples doped carbon black is in the sequence of 1.5EASSC > 3.5EASSC > 4.5EASSC, implying that the photo-generated electron-hole pairs, created in the 4.5EASSC sample, are efficiently segregated, and the 4.5EASSC sample expects to have an excellent photocatalytic activity.

### 3.3. Applied EASSC for Removal of Dye Pollutant

**Figure 6:**
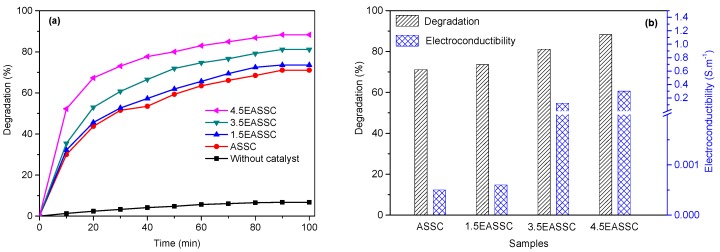
The relationship of photocatalytic degradation of basic violet 5BN dye pollutant and electrical conductivity: (**a**) Degradation; and (**b**) both degradation and electrical conductivity.

Figure 6a exhibits the photocatalytic degradation efficiency of basic violet 5BN dye in the conditions of 0.8 g of catalyst, 4 mg·L^−1^ of dye, and a UV-lamp irradiation (365 nm, 18 W) for 100 min. It can be found that the photocatalytic degradation efficiency is in the order of 88.3% (4.5EASSC) > 81.1% (3.5EASSC) > 73.6% (1.5EASSC) > 71.1% (ASSC), in which they are positive correlation to the electroconductivities of photocatalysts in Figure 6b, manifesting that the photo-generated electron enable to transmit to the network of carbon black so that the photo-generated electron and hole pairs are efficiently separated and the photocatalytic degradation efficiency is improved.

The reaction kinetics equations and relative parameters for photocatalytic degradation of basic violet 5BN dye are summarized in Table 3. The correlation coefficients of R_0_^2^, R_1_^2^, R_2_^2^, and R_3_^2^ corresponding to the zero, first, second, and third order kinetics equations. The correlation parameter is in the order of R_2_^2^ > R_3_^2^ > R_1_^2^ > R_0_^2^, respectively, implying that the photocatalytic degradation of basic violet 5BN dye belongs to the second order kinetics. The 4.5EASSC catalyst displays the best correlation of 0.99147 and the smallest half-life of 13.01 min in Table 3.

**Figure 7:**
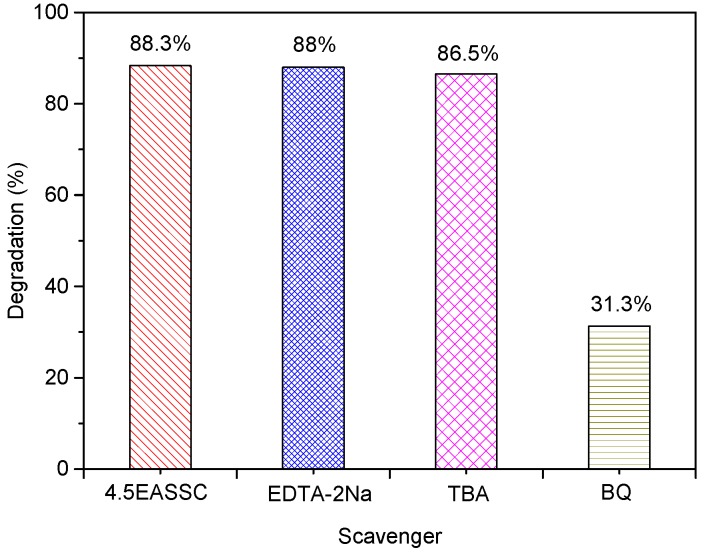
The trapping experiments of hydroxyl radical (•OH), superoxide radical (•O_2_^−^) and photogenerated hole (h^+^).

Generally, hydroxyl radical (•OH), superoxide radical (•O_2_^−^) and photo-generated hole (h^+^) are considered to be the active species in the process of photocatalytic oxidization of dye [18,19]. EDTA-2Na is usually used as a scavenger of hole (h^+^), tert-butyl alcohol (TBA) is served as a trapping agent of hydroxyl radical (•OH), and p-benzoquinone (BQ) is employed as a scavenger of superoxide radical (•O_2_^−^). From Figure 7 it is found that the degradation rate of basic violet 5BN dye over the 4.5EASSC is about 88.3% in the absence of free radical catching agent. When EDTA-2Na and TBA are separately dropped into the dye solution of basic violet 5BN suspending solid catalyst of 4.5EASSC, the degradation rates of dye slightly decrease by 0.3% and 1.8%, respectively, indicating that the active species for the degradation of dye is neither hole (h^+^) nor hydroxyl radical (•OH). After BQ is added to the reaction system of dye solution for 100 min, it is found that the degradation rate of dye sharply decreases to 31.3%, manifesting that BQ has much better inhibition effect for the degradation of dye compared to EDTA-2Na and TBA. Therefore, it is deemed to that the superoxide radical (•O_2_^−^) plays a crucial role in photocatalytic degradation of basic violet 5BN dye.

**Figure 8:**
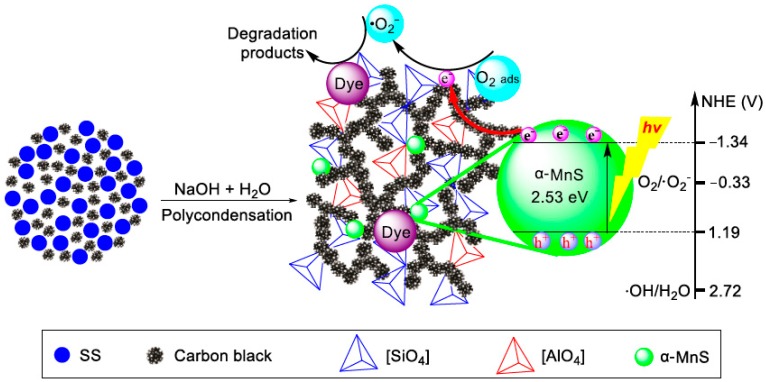
The schematic mechanism of the photocatalytic removal of dye pollutant on 4.5EASSC sample.

Figure 8 shows the schematic mechanism of the photocatalytic degradation of dye over the EASSC sample. The covalent Si–O–Si and Si–O–Al bonds in the structure of SS powders are broken to generate the monomers of sodium orthosilicate Na^+^[SiO(OH)_3_]^−^ and sodium orthoaluminate Na^+^[OAl^−^ (OH)_3_]^−^ by the alkaline activation of the aqueous NaOH solution, and then a new cementitious network structure is rebuilt by poly-condensation among monomers under the alkaline condition. The carbon black particles are simultaneously self-assembled to form the cross-linked electric-conduction networks in the interstitial sites of colloidal material, and α-MnS semiconductor disperse in the network of colloidal material. When the 4.5EASSC sample is irradiated by UV light, the electron in the valence band of α-MnS semiconductor absorbs the energy of photon which is bigger than band gap energy of 2.53 eV, and the electron jumps from the valence band to conduction band to generate both photo-induced electron (e^−^) and hole (h^+^). The positions of conduction and valence bands of α-MnS are located at −1.34 V and 1.19 V vs. NHE [20]. It is more negative than the potential of O_2_/•O_2_^−^ (−0.33 V), which indicated that the photo-excited electrons on the conduction band could reduce the adsorbed oxygen molecules to produce superoxide radical (•O_2_^−^). However, the photo-induced holes on the valence band cannot oxidize the adsorbed H_2_O molecules to form hydroxyl radical (•OH) because the valence band potential is much lower than the potential of •OH/H_2_O [21].

## 4. Conclusions

A series of electro-conductively alkali-activated silicomanganese slag-based cementitious-composites were synthesized. The electrical conductivity of EASSC samples drastically increased and the compressive strength progressive decreased with the increasing of carbon black content. The EASSC with mean grain size about 50 nm consists of (CSH), alabandite (α-MnS), and carbon black. The absorption edges of EASSC samples were gradually blue shifted with increasing carbon black content due to the literal darkening of samples, while the photo-fluorescence peak gradually decreased with increasing carbon black content due to the efficient separation of photo-induced electron-hole (e^−^/h^+^) pairs. The photocatalytic degradation efficiency of basic violet 5BN dye increased with the increasing electronic conductivities of EASSC samples, implying that the photo-generated electrons quickly transfer to the network of carbon black so that the photo-generated electrons and holes were efficiently separated and the photocatalytic degradation rate was enhanced.

## Figures and Tables

**Table 1:** Oxide composition of samples (wt %).

Sample	Na_2_O	CaO	SiO_2_	Al_2_O_3_	MgO	K_2_O	SO_3_	MnO	Fe_2_O_3_	TiO_2_	LOI
SS	0.41	21.86	28.34	18.45	4.72	1.04	2.23	11.58	0.72	0.27	10.38
4.5EASSC	5.72	18.29	26.89	17.24	4.67	1.05	2.15	11.64	0.75	0.25	11.35

**Table 2:** Electrical conductivity and compressive strength of samples.

Samples	Carbon Black (wt %)	Eectrical Conductivity at Different Curing Ages (S·m^−1^)				Compressive Strength of 28 Days (MPa)
		3 Days	7 Days	14 Days	28 Days	
ASSC	0	0.0008	0.0006	0.0005	0.0005	45.6
1.5EASSC	1.5	0.0011	0.0010	0.0007	0.0006	33.0
3.5EASSC	3.5	0.1249	0.1209	0.1169	0.1155	17.6
4.5EASSC	4.5	0.3109	0.3008	0.2976	0.2976	13.0

**Table 3:** Parameters of kinetic equations for photocatalytic degradation of basic violet 5BN dye pollutant.

Samples	Zero Order Reaction Kinetics	First Order Kinetic Equations	Second Order Reaction Kinetics Equations			Third Order Kinetics Equations
	R_0_^2^	R_1_^2^	Reaction Kinetics Equation	R_2_^2^	t_1/2_ (min)	R_3_^2^
Without catalyst	0.93534	0.93938	1/Ct = 0.000184t + 0.25221	0.9434	1357.316	0.94692
ASSC	0.76186	0.9066	1/C_t_ = 0.00619t + 0.29147	0.98656	40.78303	0.98596
1.5EASSC	0.75671	0.91077	1/C_t_ = 0.00714t + 0.29672	0.98216	35.01401	0.97857
3.5EASSC	0.70929	0.90203	1/C_t_ = 0.01112t + 0.29384	0.98965	22.48201	0.97690
4.5EASSC	0.57034	0.86509	1/C_t_ = 0.01922t + 0.32543	0.99147	13.00728	0.95239
